# Teaching and learning robotic surgery at the dual console: a video-based qualitative analysis

**DOI:** 10.1007/s11701-021-01224-5

**Published:** 2021-03-16

**Authors:** Hélène Cristofari, Minoa Karin Jung, Nadja Niclauss, Christian Toso, Laure Kloetzer

**Affiliations:** 1 Institute of Psychology and Education, University of Neuchâtel, Neuchâtel, Switzerland; 2 Division of Visceral Surgery, Department of Surgery, Geneva University Hospitals and Faculty of Medicine, Geneva, Switzerland

**Keywords:** Learning robotic-assisted surgery, Teaching robotic-assisted surgery, Dual console, Activity analysis, Video-based analysis

## Abstract

Robotic-assisted surgery (RAS) involves training processes and challenges that differ from open or laparoscopic surgery, particularly regarding the possibilities of observation and embodied guidance. The video recording and the dual-console system creates a potential opportunity for participation. Our research, conducted within the department of visceral surgery of a big Swiss, public, academic hospital, uses a methodology based on the co-analysis of video recordings with surgeons in self-confrontation interviews, to investigate the teaching activity of the lead surgeon supervising a surgeon in training at the dual console. Three short sequences have been selected for the paper. Our analysis highlights the skills-in-construction of the surgeon in training regarding communication with the operating team, fluency of working with three hands, and awareness of the whole operating site. It also shows the divergent necessities of enabling verbalization for professional training, while ensuring a quiet and efficient environment for medical performance. To balance these requirements, we argue that dedicated briefing and debriefing sessions may be particularly effective; we also suggest that the self-confrontation video technique may be valuable to support the verbalization on both the mentor’s and the trainee’s side during such debriefing, and to enhance the mentor’s reflexivity regarding didactic choices.

## Introduction

The rapid expansion of robotic surgery over the past 15 years has increased questions regarding adequate training modes for surgeons. Indeed, robotic surgery raises specific issues with regard to training, compared to open or laparoscopic procedures.

First, there has been a call for standardization both of the skills required to become an accredited surgeon [1–3], and of curricula [4]. However, assessment of a given curriculum’s efficiency is difficult. For instance, a typical approach consists of measuring “learning curves” [5–9], generally understood as the time required, or number of completed cases necessary, to acquire full command of a given procedure. As noted by Kassite et al. [10], there is no clear standard way to measure such a learning curve; moreover, some factors are rarely discussed in the robotic learning curve literature, such as patients’ outcome, intraoperative complications, the skill level of the assistant surgeon and other team members, the presence of a senior surgeon in the operating room, etc. [10].

Another difficulty is deciding who should be trained in robotic surgery [11, 12]: is it a specialty to be mastered when one is experienced in open and laparoscopic surgery, or should it be treated as a part of the general surgical toolkit, that should be familiar to everyone? This question is linked to an organizational issue, namely the presence or absence of a dedicated robotic team. Some hospitals have one team of surgeons, nurses and anesthesiologists that performs mostly robotic interventions, which enables them to become highly specialized. Other institutions do not have dedicated robotic teams; this decreases exposure to robotic surgery [13, 14], and makes it more difficult to maintain expertise and train new surgeons in robotic procedure completion. In fact, many have argued that a sufficient volume of cases is a prerequisite for an adequate robotic training and stable level of competence [15, 16].

Another important constraint on training is the need to minimize the operating time: some robotic procedures may already take longer than open surgery. Hence, it is paramount that operative time is not extended further by moments of teaching that slow down the procedure [17, 18]. This creates a tension between the need to train some of the operating team, and the need to perform as efficiently as possible.

Robotic surgery also radically transforms the operating room’s spatial configuration, creating a distance between the lead surgeon at the console and the other operating team members at the bedside or the second console. It has been suggested that this distance may impede effective teaching [19], particularly by hampering non-verbal communication.

Training may be hindered by financial restrictions, limiting access to useful tools. A dual console that may be used to operate with a novice is very costly, and many studies evaluating the usefulness and validity of simulators emphasize the issue of their cost [20–24].

Finally, collaboration modes are transformed during robotic intervention. The lead surgeon requires less help from trainees (such as holding retractors in open surgery), thus diminishing the opportunity for active peripheral participation [11, 25]. This limited involvement of trainees, which led Hanly et al. [26] to describe robotic surgery as “resident-unfriendly,” might explain a survey which found that nearly half of residents believe robotic cases interfere with their training [27].

Very few studies have investigated training as it happens in the operating room. Exceptions include Beane’s study [11], which argued that residents develop skills in robotic surgery through “shadow learning,” a set of learning practices that might not be safe nor adequately integrated in their broader curriculum, leading to “undersupervised struggles” (e.g., performing at the edge of one’s skills with poor supervision).

Beyond these difficulties, robotic surgery offers two major opportunities for operating room training: the dual-console system and the automatic video-recording of operations. As described by Fernandes et al. [28], the dual console on the da Vinci robotic surgical system (Intuitive Surgical, Sunnyvale Inc.) enables proctoring; for instance, the lead surgeon may give full control to the trainee, or give control over two arms but retain control over the third one to gradually ease the trainee into the more complex steps of the procedure, or may indicate particular locations with the aid of pointers. Goonewardene et al. [29] emphasized the didactic potential of these functions, stating that “*dual-console training allows for unique trainee–trainer dynamics that are simply not possible with single-console robotics or even open surgery*.”

It thus appears crucial to make the most of video and of the dual-console system, given the otherwise reduced opportunities for participation in actual robotic procedures. What remains missing, however, is an analysis of how the didactical functions of the dual console are mobilized by the surgeon to train novices in robotic surgery, and how such training may be further enhanced. Therefore, our goals are to shed light on surgeons' training at the dual console as it happens during actual surgeries, and to identify didactic difficulties that may be resolved with targeted effort.

## Method

Our study was conducted in a Swiss university hospital, within the visceral surgery unit. We focused on one specific robotic procedure, namely the robotic Roux-en-Y gastric bypass. This operation was selected by the surgeons as a standardized intervention that follows well-defined, precise steps (summarized in Table 1 below). It was, therefore, considered ideal for training purposes. This hospital has access to two da Vinci Xi robotic systems, used in urology, gynecology and visceral surgery. The gastric bypasses are performed by a senior surgeon and a trainee. For these interventions, the regular operating team consists of a senior surgeon operating at the console, an assistant surgeon at the bedside, a bedside nurse, a circulating nurse, assistant nurses for opening and closing the operating room, and the anesthetic team. Trainees may also be present.

**Table 1:** Approximate timeline of the gastric bypass

Steps of the gastric bypass	Approximate duration
Skin incision, positioning of ports, diagnostic laparoscopy, liver retraction, and docking	10 min
Creation of stomach pouch (stapling of stomach to create a gastric pouch)	15 min
Gastrojejunal anastomosis (suturing gastric pouch with jejunal limb)	25 min
Bowel measurement	5 min
Jejunojejunal anastomosis (suturing two segments of the bowel)	20 min*
Closure of mesenteric defects	10 min
Leakage check	5 min
Dedocking and skin suture	10 min

*The selected excerpts

Our research involved three field-work phases.

*Phase 1: Observations and interviews *From July to October 2019, we observed ten robotic gastric bypasses. We took notes focusing on each participant’s technical gestures, and on the communication between the surgical team members. We conducted four formal interviews with surgeons (consultants, staff surgeons and residents), as well as a number of informal, brief follow-up interviews. These interviews aimed to ascertain the practitioners’ interpretation of the events observed, and provide a broad view of the surgeons’ training pathway with regard to robotic surgery, including the difficulties encountered and questions raised.

*Phase 2: Filming *From November 2019 to January 2020, we filmed three gastric bypasses, from the operating room set-up and patient’s preparation, to the surgeons’ departure. These three interventions differed in their configuration of surgical staff, as summarized below:

*Bypass 1:* Surgical staff included a lead surgeon, a bedside assistant fully qualified as a staff surgeon and already advanced in robotic training at the second console, and a resident with little experience in robotic surgery. The staff surgeon assisting bedside left the bedside to observe and operate at the second console around the middle of the intervention, and at this point the resident assumed the bedside assistant role.

*Bypass 2:* This was attended by a lead surgeon, a bedside assistant fully qualified as a staff surgeon but with almost no robotic surgery experience, and a resident surgeon at the beginning of their residency.

*Bypass 3:* Those present included a lead surgeon, and a bedside assistant fully qualified as a staff surgeon and already advanced in robotic training at the second console (as in bypass 1). The lead surgeon and bedside assistant switched roles between the console and bedside position around the middle of the procedure, for training purposes.

All three surgeries were integrally transcribed.

**Fig. 1:**
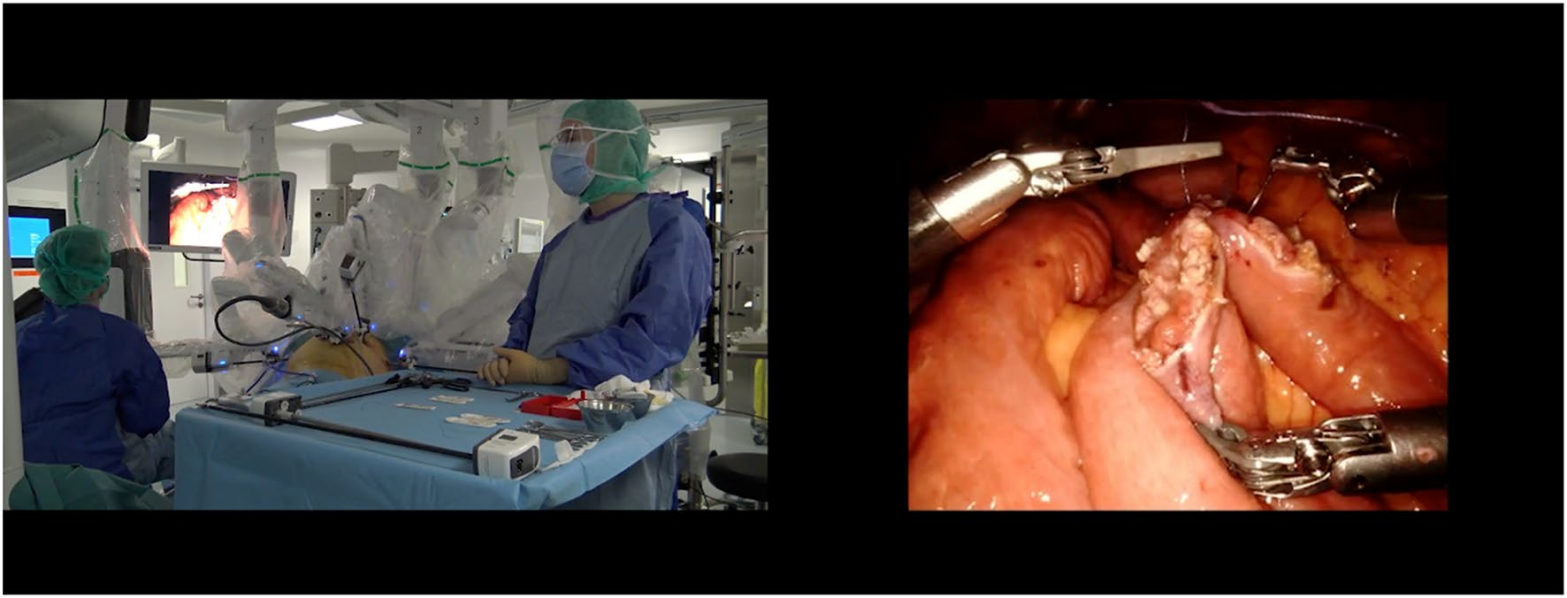
Montage of the robot’s video synchronized with the film of the operating room

*Phase 3: Self-confrontation interviews *A montage was created for each robotic Roux-en-Y gastric bypass, synchronizing the audio and video-recording of the operating room and the images recorded by the robot’s camera inside the patient’s abdomen, to enable simultaneous viewing of the operating room and inside the patient’s body (Fig. 1).

Edited excerpts of the montages were subsequently used to conduct video-based self-confrontation interviews [30, 31] with the lead surgeon and two assistant surgeons. This self-confrontation technique, which has been fruitfully implemented to study robotic expertise [32], consists of showing the practitioner a video of themselves at work and asking them to comment on it to understand their take on their own activity, while also fostering reflexivity.

During these self-confrontation interviews, three excerpts were selected, in discussion with the lead surgeon, to be more closely analyzed. These excerpts reflect some interesting moments of teaching at the second console or the bedside. They happen during the jejunojejunal anastomosis (see Table 1 below).

## Results

### Situation 1: communication in training


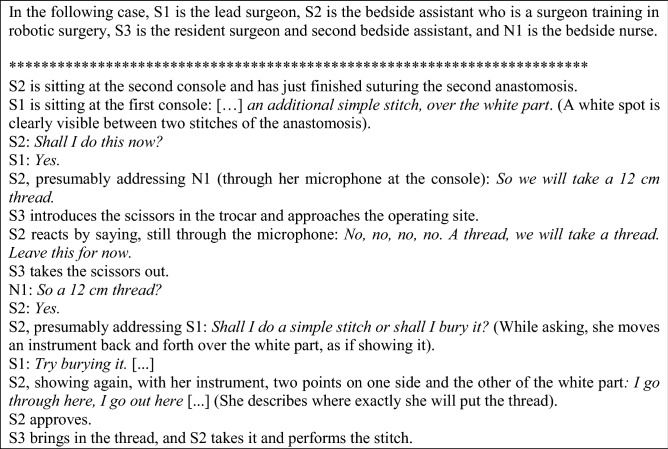


In this short excerpt, we would like to highlight two micro-events. First, S1’s supervision here implies directive speech acts and limited communication. S1 asks S2 to perform “*an additional simple stitch”* without further explanation; she only specifies the location (“*on the white part*”). In the self-confrontation interview, S1 commented that explanations on her underlying rationale are unnecessary.

In a follow-up interview, S2 indicated that the “*white part*” was a cauterized region of the bowel which should end up inside the suture, as when it remains outside, the risk of leak from that point is higher. Thus, S2 was able to make sense of S1’s request for an additional stitch, which may appear in line with S1’s belief that further explanations would have been unnecessary. However, S2 does not take this initiative during the intervention, and she also appears to doubt the correct moment to proceed (“*shall I do this now?*”), as well as the technique to be used (a simple stitch or a buried one). S2 also asks for confirmation regarding the details of the gesture, using the graspers to show the location: the technical tools are used here with a communicative function. These questions raise the issue of whether more explanations about this step might have been useful for S2’s training. Such discussions may happen after the surgery, as mentioned in the discussion part of this article.

The procedure is further disrupted because of a mistake made by the bedside assistant, when S2 stops the assistant from wrongly introducing the scissors. As explained by S1 in the self-confrontation interview, “*this is very understandable, why the bedside assistant put the scissors in* […]. *He put the scissors in because* [S2] *is holding the thread to stabilize the anastomosis. And he interprets it as a request to cut the thread. That’s why, for him it’s the visual that tells him “ah, I must cut this thread because she’s presenting it to me,” and at the same time I’m asking for a thread for S2 to make the stitch, so it is two divergent pieces of information*.” Here, the mistake comes from the differences of robotic surgery compared to traditional surgeries (holding the thread to stabilize the anastomosis versus a request to cut it). Hence, the mistake is potentially due to the fact that S3 is a novice, and because S2 has not communicated the requirements clearly enough.

Managing the bedside is in fact a skill that one must develop through training at the second console, both regarding the verbal communication and the coordination of gestures. As detailed by S1: “*What I find interesting is that we can also* […] *present the thread so that he can take it, or we can* […] *just hold it, and* [S2] *does it very often, she does not present it, she does not make life easier for the bedside assistant, and that’s also a sign*…*. It’s like when you’re driving and you have to make things easy for people close by, for people further away. And* [S2] *is often very concentrated on what she’s doing, but not yet on how to make the whole team work well… It’s not a reproach, it’s just a comment I’d say; these are things that will get better over time*.” The moment in which this operation was recorded (with S2 being in the middle of robotic surgery training) enables us to see robotic skills in development: S2 has already gained the skills to manage her own actions in this part of the surgery, but not yet the skills of managing the whole team’s work.

**Fig. 2:**
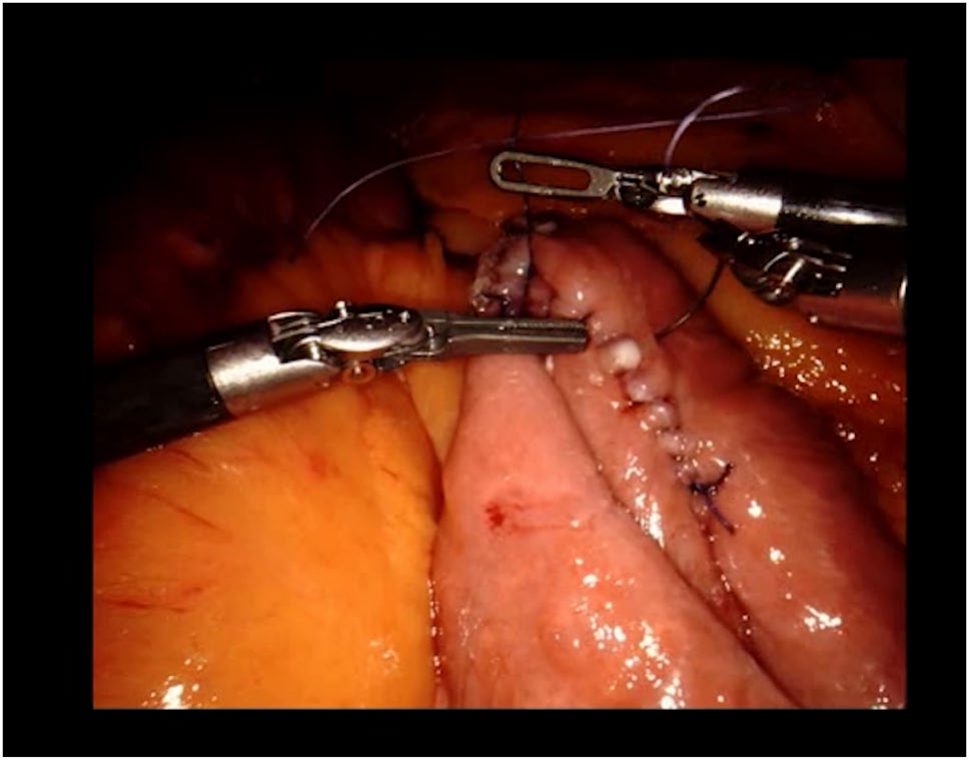
Adding a supplementary stitch in the second anastomosis

Managing novice members of the whole team creates an additional mental load for the surgeon. S1 commented that when the bedside assistant has *“almost zero experience at the robot* […]*, I am busier watching the bedside. Because just by changing an arm, a bedside assistant with little experience may perforate the liver for instance*.” That’s why “*The ideal situation is with a experienced staff surgeon assisting* [at the second console] *with the FMH title* [i.e. holder of the Swiss “Foederatio Medicorum Helveticorum” title] *and a bedside assistant already trained in robotics who does not yet have the FMH title who is at the bedside. It is this situation which allows me to concentrate on the one at the second console and stop watching outside the console what the bedside assistant is doing*.” In fact, watching S2 at the second console is already demanding for S1, as she insists that taking control back is not instantaneous: you need a few seconds to change the parameters on the console’s touchscreen, and these seconds may be crucial. A novice at the bedside is thus a heavy load. However, the ideal teaching situation described by S1 is far from being the general rule, and teaching at the second console may even happen in the absence of a third surgeon, with S1 performing the role of the bedside assistant, as is the case in the two situations detailed hereafter (Fig. 2).

### Situation 2: learning to work faster, using two or three hands

In this case, S1 is the lead surgeon (same as in example 1), who has left the first console and is assisting at the bedside; S2 is the staff surgeon training in robotic surgery (same as in example 1), who is now operating from the second console. There is no third surgeon present.

**Fig. 3:**
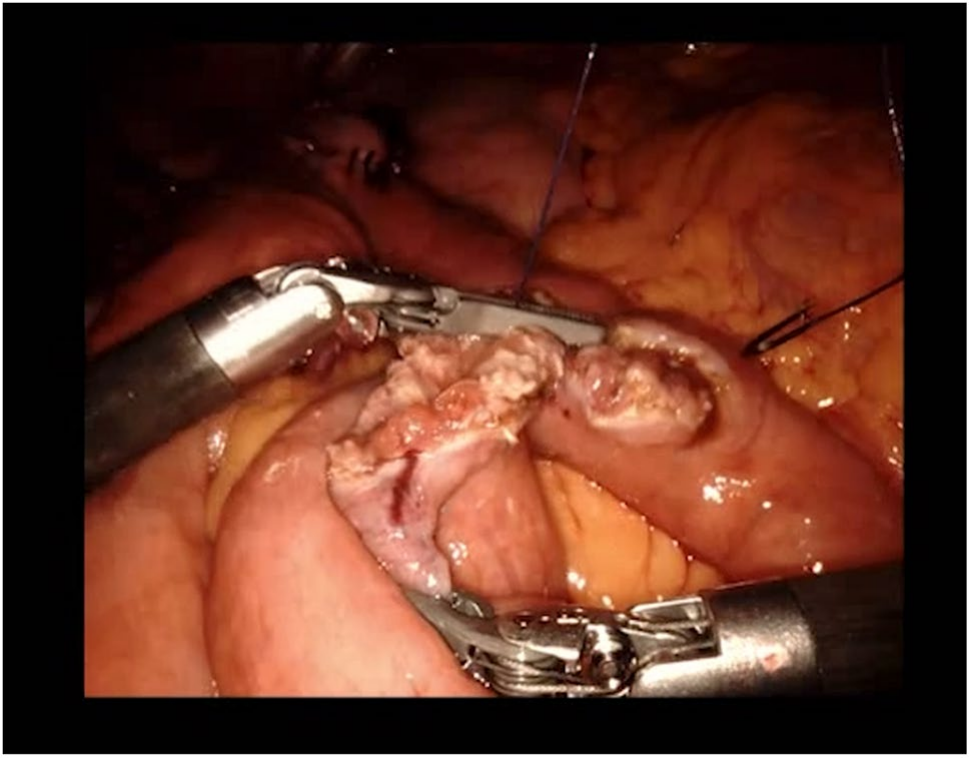
Ensuring correct tension of the thread during the second anastomosis

S2 has taken control at the second console from the beginning of the second anastomosis, and is now finishing suturing the posterior intestinal wall. To obtain an adequate tension, S2 holds the thread up, statically, with the right hand, while pushing the bowel downwards with the left hand (Fig. 3).

In the self-confrontation interview, S1 commented on this particular gesture, saying that it is something S2 has to make progress in: “*when she has to tighten the stitch, it’s sometimes a bit static. She uses the left hand to push the bowel downwards, she holds the thread up, but she should actively tighten it upwards at the same time; it’s a bi-manual movement. And functioning in a bi-manual way, it’s something that develops, or even three hands here controlling three robotic arms in addition to the camera arm, it develops with experience. Often at the beginning you use only one hand really actively, the other one remains a bit stable*.”

This use of both hands to dynamically tighten the suture appears to reflect a major challenge for trainees in robotic surgery, namely making adequate use of the four arms of the robot, as explained by S1: *“That’s a very good question, it only strikes me now. The difference between robotic and open surgery is that the main surgeon is at the same time her own assistant, which is not the case for open surgery. In open surgery, you would have an assistant, a resident who holds the thread for you* […]. *In open surgery, you don’t have three hands, only two* […], *so it’s another person who holds the thread for you.* […]. *In robotic surgery you are your own assistant* […]. *That’s one of the additional challenges you encounter in robotic surgery. And, moreover, you also have to manage the camera, which you don’t have to do in open surgery. You have a clutch at your feet to direct the camera* […]*, compared to laparoscopic surgery where you have still another assistant to move the camera for you; as you have only two hands in laparoscopic surgery, neither a third one for the camera nor a third one for a third instrument*.”

This difficulty of adequate management of the third arm may also create other more pressing issues, such as instrument collision, as will be exemplified hereafter.

### Situation 3: perceiving the whole operating field

This situation happened just a few minutes after the previous one. S1, who remains the lead surgeon, has left the first console and is assisting at the bedside; and S2, the surgeon training in robotic surgery, is now operating from the second console. S2 is continuing the suture of the second anastomosis.

**Fig. 4:**
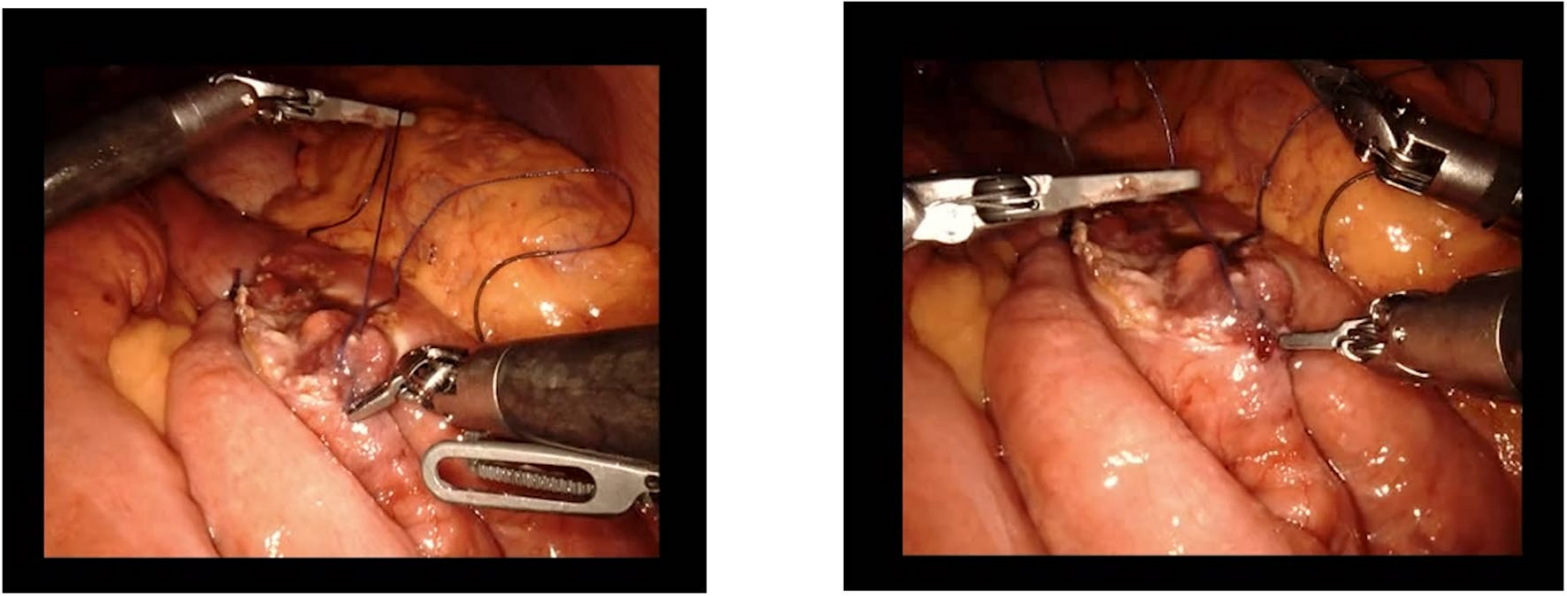
Managing adequate placement of the three instruments during the second anastomosis: **a** collision with the fourth arm’s instrument; and **b** misplacement of an instrument

S2 is using two pliers to manipulate the thread and needle, and the third arm, also equipped with pliers, is left immobile in the operating site, very close to the actively used instruments. As she is suturing, S2 lets her left hand’s pliers touch the third instrument arm, and press on it for a couple of seconds (Fig. 4a). S1, from the bedside, draws her attention to it (“careful with the third arm”). S2 stops the instruments’ collision, and moves the third arm away, higher up. She places it with the pliers themselves creating an angle with the arm, with this angle pointing towards the operating site (Fig. 4b), and leaves it immobile, while she continues suturing. S1 makes no other comment.

In the self-confrontation interview, S1 refers to the collision: “*You see how there is a collision between these two instruments* […] *and it damages the instruments, when you have that. Because the strength of this arm is such, that it damages the other one* […] *and these things are to be avoided, such collision between instruments”* According to S1, collisions happen mostly not because of a handling difficulty, but because of a lack of adequate perception, which is a skill developed when training at the second console: *“that is also, probably, one of the most difficult things at the beginnings with the robot: seeing everything. It’s like when you’re driving a car, and you have to watch not only the road in front of you but also the other cars, the one that is leaving the parking lot… seeing the whole of the operating field, and not only concentrating on “ok now I must make a stitch here with these two hands that I have here,” but also seeing the damage that is being done over there.”*

After S1 makes S2 aware of the collision, S2 moves the instrument away, but that is not done in an optimal way, as specified by S1: *“now it’s in a good spot, but you see the angle, it can still bother us, there can still be a collision.* […]; *the instrument should be angled the other way* […] *pointing away from the operating site.* […]. *It’s quite detailed, but it’s such little things that make the process less efficient*.”

These two issues—instrument collision and instrument misplacement—are addressed differently by S1 during the surgery. In the case of instrument collision, “*Usually I tell* [the trainee at the second console] *when this happens, let’s see… *[…]. *Yeah, I said something like “careful with the third arm”* […] *because she doesn’t see it.*[…] *I tell her to take it away; she takes it away, she puts it on the side* […].” However, when S2 misplaces the third arm’s instrument, S1 does not make any comment.

This choice may not always be understandable to S2, as expressed by S1:*“We’ve had moments of tension with* [the trainee]*; she would ask “why don’t you tell me”* […], *but things that I say 10 or 20 or 30 times, one day I will stop saying them.* […] *It’s not my job to tell it over and over again* […]. *In my opinion, the person won’t make progress, but that’s just my opinion, she will always wait for the comment, and correct it only when you say it. But one day she must feel by herself what happens if she doesn’t do this or that, otherwise there’s no progress. Some comments are crucial for the patient’s outcome* […] *and you will repeat them until your last day, that’s for sure. But such things* [as the inadequate angle of the instrument] *make the operating site less convenient* […], *but there’s absolutely no danger for the patient, it’s only bothering*.” Thus, S1 expresses her didactic theory, which implies correcting the trainee when the error may have important consequences for the surgical outcome, but letting the trainee experience the consequences of the minor mistakes, so that she will gain autonomy instead of relying permanently on S1’s corrections.

## Discussion

Overall, our results indicate that verbal communication between the lead surgeon and the trainee at the second console is focused and limited. For the safety of the patient, quick and efficient gestures and team coordination are needed, which should ideally not require too much communication, as expressed by the lead surgeon: “*The ideal surgery is wordless*.” This appears at odds with a major principle of professional didactics: Pastré [33] argues that professional development involves becoming conscious of one's implicit knowledge through a process of *conceptualization*, and highlights the central roles of *dialogue* and *verbalization* in professional training to enable such a conceptualization. Furthermore, the physical separation between the lead surgeon and the trainee means that some non-verbal communication such as eye-contact or bodily contact may be lacking, which makes it harder for the lead surgeon to adequately correct the trainee and provide feedback on the trainee’s ongoing performance. This lack of embodied guidance makes adequate verbal communication even more necessary. Consequently, professional training and medical performance may create divergent challenges and requirements, which will be further discussed here.

The importance of verbalization as revealed in professional didactics is also visible in our results. Indeed, our analyses show the usefulness of verbalizing a planned action before performing it: it enables the trainee to make their planned action explicit and get confirmation of the specific gestures to be accomplished, thus also enabling the lead surgeon to correct the verbalization instead of the potentially inadequate decision itself. This may be particularly relevant for robotic surgery, as the potentially low volume of cases reinforces the need for the trainees to make the most of every case they participate in.

Our data show that the lead surgeon briefly comments on immediate actions to be accomplished in the specific context of the operation, but does not expand on more generic principles of action, nor do they provide explanations about the underlying rationale behind a particular decision. Furthermore, our results show that the trainee rarely asks questions that go beyond asking for immediate confirmation of a specific gesture. Exploring the consequences of the physical separation between the lead surgeon and the team, El-Hamamsy et al. [19] suggest that this separation may also be an obstacle to asking questions, as trainees tend to find this distance intimidating. Molloy and Bearman [34] offer a complementary explanation: looking more broadly at health profession education, they emphasize the need for “*intellectual candor*”, defined as a way to embrace the “*tension between expressing vulnerability and appearing credible*.” During robotic surgery, asking the lead surgeon a question requires the trainee to express this vulnerability or potential lack of skills in a way that must be heard by the lead surgeon, but also most of the team. The physical separation may thus further enhance the emotional difficulties associated with asking questions.

We might also suggest a third and complementary direction to understand this limited communication: the surgeons already work at a very high level of mental load, having to manage the patient’s safety in the operating process, their own demanding actions (like working with three hands), the whole team’s actions (such as controlling the novice bedside assistant’s actions), and communication with the whole team. Therefore, additional questions by the surgeon in training or comments by the lead surgeon might appear as sources of disruption for both surgeons.

Indeed, this tension between the need to allow for verbalization as part of the process of conceptualization in action, and the need to alleviate the pressure of interaction weighing on the lead surgeon in particular, appears to be the crux of the issues surrounding training in the operating room. Previous research has shown the ambivalent effect of verbal exchanges during surgeries. Tschan et al. [35] thus suggest that “case-relevant” communication can enhance a patient’s outcome, and that “case-irrelevant” communication may impair surgeons’ concentration during certain surgical steps, while also fostering team cohesiveness and collaboration.

When the lead surgeon is responsible for teaching a trainee, the need for flexible adaptation to both the surgery in progress and the surgeon in training requires making sense of sensorial cues and integrating them with previous knowledge and experience. Moreover, in the case of robotic surgery, the lead surgeon must rely mostly on limited visual and auditory information to understand the patient’s situation and the course of the operation, make medical decisions, and monitor the “atmosphere” of the team (including the trainee’s needs, capabilities, and emotional confidence). We have also discussed the fact that the lead surgeon must do without the more implicit dimensions of training such as side-by-side observation and embodied guidance, which are substituted with explicit verbal indications and pointing gestures on the screen to direct the trainee’s attention and actions. By reducing the communication channels that surgeons can use for conducting the surgery and supervising trainees, robotic surgery hence creates an additional mental load for the lead surgeon, thus making it even more important to maintain a quiet environment and reduce disruptions as much as possible.

Improvements in the sound-transmission system of the robot, as well as improvements in the set-up of the operating room, may help in alleviating some of these communication issues. In particular, the sound transmission between the lead surgeon’s console and the team at the bedside could benefit from a redesign to ensure smoother communication in both directions; while a revised set-up of the room would help in ensuring an easier visual contact between the surgeon at the lead console, the trainee at the second console, and the bedside team.

However, such material improvements may only partly address the issue at stake, and we remain with an apparent paradox between fostering dialogue to enhance professional training and maintaining a quiet environment to enable concentration. A potential resolution involves enhancing the discussion and verbalization of experts’ and trainees’ thought processes outside of the operating room. This would require dedicated briefing and debriefing time, which is also commonly highlighted in professional didactics [36]. A lack of such briefing and debriefing has been pointed out by surgeons as a major issue for training. Porte et al. [37] and O’Connor et al. [38] quantitatively assessed the impact of expert feedback on the development of surgical skills, and found a significant positive effect. Champagne [39] argues that “*Quality teaching moments will only be realized when emphasis is placed on preparation, useful instruction during the procedure, and postoperative feedback.*” Previous work also indicates that the self-confrontation technique, which was used here to investigate the teaching processes, is useful not only as a research tool to explore training issues, but also as a training tool in itself to foster reflexivity [32, 40]; it thus appears to be a promising tool for debriefing, to enhance trainees' and trainers' conceptualizations following robotic procedures.

## Conclusion

The robotic operating room organization involves training processes that differ from open or laparoscopic surgery, particularly regarding the possibilities of observation and embodied guidance. The dual-console system creates a potential opportunity for participation. To make the most of this didactic potential, careful attention must be given to the divergent necessities of enabling verbalization for professional training, while ensuring a quiet and efficient environment for medical performance. To balance these requirements, we argue that dedicated briefing and debriefing sessions may be particularly effective; we also suggest that the self-confrontation video technique may be valuable to support the verbalization on both the mentor’s and the trainee’s side during such debriefing, and to enhance the mentor’s reflexivity regarding didactic choices.

Our analysis focuses on the interactions between the lead surgeon and the trainee at the dual console, but the collaboration and didactic interactions also involve other members of the operating team, the bedside assistant in particular. We thus suggest that a next useful step would consist of further analysis of the bedside surgeon’s role in the robotic training process.

## References

[1] Lee JY, Mucksavage P, Sundaram CP, McDougall EM (2011). Best practices for robotic surgery training and credentialing. J Urol.

[2] Dulan G, Rege RV, Hogg DC, Gilberg-Fisher KM, Arain NA, Tesfay ST, Scott DJ (2012). Developing a comprehensive, proficiency-based training program for robotic surgery. Surgery.

[3] Smith R, Patel V, Satava R (2014). Fundamentals of robotic surgery: a course of basic robotic surgery skills based upon a 14-society consensus template of outcomes measures and curriculum development. Int J Med Robot.

[4] Schreuder HW, Wolswijk R, Zweemer RP, Schijven MP, Verheijen RH (2012). Training and learning robotic surgery, time for a more structured approach: a systematic review. BJOG.

[5] Heemskerk J, van Gemert WG, de Vries J, Greve J, Bouvy ND (2007). Learning curves of robot-assisted laparoscopic surgery compared with conventional laparoscopic surgery: an experimental study evaluating skill acquisition of robot-assisted laparoscopic tasks compared with conventional laparoscopic tasks in inexperienced users. Surg Laparosc Endosc Percutan Tech.

[6] Lenihan JP, Kovanda C, Seshadri-Kreaden U (2008). What is the learning curve for robotic assisted gynecologic surgery?. J Minim Invasive Gynecol.

[7] Lee J, Yun JH, Nam KH, Soh EY, Chung WY (2011). The learning curve for robotic thyroidectomy: a multicenter study. Ann Surg Oncol.

[8] Buchs NC, Pugin F, Bucher P, Hagen ME, Chassot G, Koutny-Fong P, Morel P (2012). Learning curve for robot-assisted Roux-en-Y gastric bypass. Surg Endosc.

[9] Vilallonga R, Fort JM, Gonzalez O, Caubet E, Boleko A, Neff KJ, Armengol M (2012). The initial learning curve for robot-assisted sleeve gastrectomy: a surgeon’s experience while introducing the robotic technology in a bariatric surgery department. Minim Invasive Surg.

[10] Kassite I, Bejan-Angoulvant T, Lardy H, Binet A (2019). A systematic review of the learning curve in robotic surgery: range and heterogeneity. Surg Endosc.

[11] Beane M (2019). Shadow learning: building robotic surgical skill when approved means fail. Admin Sci Q.

[12] Ismail A, Wood M, Ind T, Gul N, Moss E (2020). The development of a robotic gynaecological surgery training curriculum and results of a Delphi study. BMC Med Educ.

[13] Kang MJ, De Gagne JC, Kang HS (2016). Perioperative nurses’ work experience with robotic surgery: a focus group study. Comput Inform Nurs.

[14] Gillespie BM, Gillespie J, Boorman RJ, Granqvist K, Stranne J, Erichsen-Andersson A (2020). The impact of robotic-assisted surgery on team performance: a systematic mixed studies review. Hum Factors.

[15] Cuschieri A (2003). Lest we forget the surgeon. Semin Laparosc Surg.

[16] Carlos G, Saulan M (2018). Robotic emergencies: are you prepared for a disaster?. AORN J.

[17] Hyun MH, Park JW, Shin DS, Cho JM, Yang KS, Park S (2014). Minimizing operative time for robotic gastrectomy in cancer: analysis of the major factors for four detailed steps. Hepatogastroenterology.

[18] Lee JM, Yang SY, Han YD, Cho MS, Hur H, Min BS, Lee KY, Kim NK (2020). Can better surgical outcomes be obtained in the learning process of robotic rectal cancer surgery? A propensity score-matched comparison between learning phases. Surg Endosc.

[19] El-Hamamsy D, Walton TJ, Griffiths TRL, Anderson ES, Tincello DG (2020). Surgeon-team separation in robotic theaters: a qualitative observational and interview study. Female Pelvic Med Reconstr Surg.

[20] Sethi AS, Peine WJ, Mohammadi Y, Sundaram CP (2009). Validation of a novel virtual reality robotic simulator. J Endourol.

[21] Bric J, Connolly M, Kastenmeier A, Goldblatt M, Gould JC (2014). Proficiency training on a virtual reality robotic surgical skills curriculum. Surg Endosc.

[22] Bric JD, Lumbard DC, Frelich MJ, Gould JC (2016). Current state of virtual reality simulation in robotic surgery training: a review. Surg Endosc.

[23] Ibrahim AM, Varban OA, Dimick JB (2016). Novel uses of video to accelerate the surgical learning curve. J Laparoendosc Adv Surg Tech A.

[24] Bresler L, Perez M, Hubert J, Henry JP, Perrenot C (2020). Residency training in robotic surgery: the role of simulation. J Visc Surg.

[25] Zhao B, Hollandsworth HM, Lee AM, Lam J, Lopez NE, Abbadessa B, Eisenstein S, Cosman BC, Ramamoorthy SL, Parry LA (2020). Making the jump: a qualitative analysis on the transition from bedside assistant to console surgeon in robotic surgery training. J Surg Educ.

[26] Hanly EJ, Miller BE, Kumar R, Hasser CJ, Coste-Maniere E, Talamini MA, Aurora AA, Schenkman NS, Marohn MR (2006). Mentoring console improves collaboration and teaching in surgical robotics. J Laparoendosc Adv Surg Tech A.

[27] Farivar BS, Flannagan M, Leitman IM (2015). General surgery residents’ perception of robot-assisted procedures during surgical training. J Surg Educ.

[28] Fernandes E, Elli E, Giulianotti P (2014). The role of the dual console in robotic surgical training. Surgery.

[29] Goonewardene SS, Brown M, Challacombe B (2016). Single- versus dual-console robotic surgery: dual improves the educational experience for trainees. World J Urol.

[30] Clot Y, Faïta D, Fernandez G, Scheller L (2000) Entretiens en autoconfrontation croisée: une méthode en clinique de l’activité. Perspectives Interdisciplinaires Sur Le Travail et la Santé 146(2–1)

[31] Kloetzer L, Clot Y, Quillerou-Grivot E, Filliettaz L, Billett S (2015). Stimulating dialogue at work: The activity clinic approach to learning and development. Francophone perspectives of learning through work.

[32] Seppänen L, Kloetzer L, Riikonen J, Wahlström M (2016). A developmental perspective to studying objects in robotic surgery. Working conference on information systems and organizations.

[33] Pastré P (1999). La conceptualisation dans l'action: bilan et nouvelles perspectives. Educ Permanente.

[34] Molloy E, Bearman M (2019). Embracing the tension between vulnerability and credibility: ‘intellectual candour’ in health professions education. Med Educ.

[35] Tschan F, Seelandt JC, Keller S, Semmer NK, Kurmann A, Candinas D, Beldi G (2015). Impact of case-relevant and case-irrelevant communication within the surgical team on surgical-site infection. Br J Surg.

[36] Pastré P, Mayen P, Vergnaud G (2006). La didactique professionnelle. Revue française de pédagogie. Recherches en Education.

[37] Porte MC, Xeroulis G, Reznick RK, Dubrowski A (2007). Verbal feedback from an expert is more effective than self-accessed feedback about motion efficiency in learning new surgical skills. Am J Surg.

[38] O'Connor A, Schwaitzberg SD, Cao CG (2008). How much feedback is necessary for learning to suture?. Surg Endosc.

[39] Champagne BJ (2013). Effective teaching and feedback strategies in the OR and beyond. Clin Colon Rectal Surg.

[40] Seppänen L, Schaupp M, Wahlström M (2018). Enhancing learning as theoretical thinking in robotic surgery. Nordic J Vocational Educ Training.

